# Predictors of pulmonary sequelae after COVID-19 pneumonia: A 12-month follow-up study

**DOI:** 10.3389/fmed.2023.1084002

**Published:** 2023-02-02

**Authors:** Nicol Bernardinello, Elisabetta Cocconcelli, Chiara Giraudo, Matteo Daverio, Gioele Castelli, Simone Petrarulo, Matteo Bovo, Giulia Fichera, Silvia Cavinato, Anna Maria Cattelan, Marina Saetta, Paolo Spagnolo, Elisabetta Balestro

**Affiliations:** ^1^Respiratory Disease Unit, Department of Cardiac, Thoracic, Vascular Sciences and Public Health, University of Padova and Padova City Hospital, Padova, Italy; ^2^Department of Medicine, Institute of Radiology, University of Padova and Padova City Hospital, Padova, Italy; ^3^School of Medicine and Surgery, University of Padova, Padova, Italy; ^4^Pediatric Radiology Unit, University of Padova and Padova City Hospital, Padova, Italy; ^5^Division of Infectious and Tropical Diseases, University of Padova and Padova City Hospital, Padova, Italy

**Keywords:** COVID-19, pulmonary fibrosis, 12-month follow-up, CT scan, SARS-CoV-2

## Abstract

**Background:**

Since the beginning of the SARS-CoV-2 pandemic, over 550 million people have been infected worldwide. Despite these large numbers, the long-term pulmonary consequences of COVID-19 remain unclear.

**Aims:**

The aim of this single-center observational cohort study was to identify and characterize pulmonary sequelae of COVID-19 at 12 months from hospitalization and to reveal possible predictors for the persistence of long-term lung consequences.

**Methods:**

Based on the persistence or absence of radiological changes after 12 months from hospitalization, the whole population was categorized into NOT-RECOVERED (NOT-REC) and RECOVERED (REC) groups, respectively. Clinical and pulmonary function data tests and clinical data were also collected and compared in the two groups. In the NOT-REC group, high resolution computed tomography (HRCT) images were semiquantitatively scored analyzing ground-glass opacities (GGO), interstitial thickening (IT), consolidations (CO), linear and curvilinear band opacities, and bronchiectasis for each lung lobe. Logistic regression analyses served to detect the factors associated with 12-month radiological consequences.

**Results:**

Out of the 421 patients followed after hospitalization for SARS-CoV-2 pneumonia, 347 met inclusion and exclusion criteria and were enrolled in the study. The NOT-REC patients (*n* = 24; 6.9%) were significantly older [67 (62–76) years vs. 63 (53–71) years; *p* = 0.02], more frequently current smokers [4 (17%) vs. 12 (4%); *p* = 0.02], and with more severe respiratory failure at the time of hospitalization [PaO_2_/FiO_2_ at admission: 201 (101–314) vs. 295 (223–343); *p* = 0.01] compared to REC group (*n* = 323; 93.1%). On multivariable analysis, being a current smoker resulted in an independent predictor for lung sequelae after 12 months from hospitalization [5.6 OR; 95% CI (1.41–22.12); *p* = 0.01].

**Conclusion:**

After 12 months from hospital admission, a limited number of patients displayed persistent pulmonary sequelae with minimal extension. Being a current smoker at the time of SARS-CoV-2 infection is an independent predictive factor to lung consequences, regardless of the disease severity.

## Introduction

SARS-CoV-2 has spread quickly around the world since December 2019, infecting hundreds of millions of people. Despite our knowledge about this virus constantly growing, the complete understanding of long-term complications remains unclear (1). The clinical course of COVID-19 could be highly heterogeneous, in some cases with severe respiratory complications, necessitating an intensive care unit hospitalization (ICU) (2). Moreover, a percentage of patients after the acute phase could develop long-term complications of the virus, such as chronic fatigue, dyspnea, brain fog, muscle dizziness, and other neurocognitive conditions, reducing quality of life and daily activity tasks (3, 4). Previous SARS and MERS epidemics demonstrated that symptoms and imaging abnormalities persist over time, hence, it has been suggested to monitor patients after acute SARS-CoV-2 pneumonia (5). In 2020, the British Thoracic Society (BTS) produced a document for post-COVID-19 management, distinguishing severe pneumonia and patients with mild-moderate pneumonia (6). The purpose of this document was to standardize radiological follow-up and then mitigate the pressures on respiratory services after the initial COVID-19 outbreak. Thus, in these last 2 years, more effort was spent to identify specific clinical and biological attributes before and during COVID-19 infection that can be predictive of which symptoms and clinical course patients may develop (7). Other studies tried to investigate, both in severe and non-severe ill patients, the prevalence and the risk factor of pulmonary fibrosis after COVID-19 infection. Several authors reported a percentage of 19% at 4 months (8), while others reported a higher prevalence at 7 and 12-month follow-ups (9–11). However, few data have been published concerning a longer observational period. Thus, the aim of this study was to identify and characterize, among patients hospitalized for SARS-CoV-2 infection, those exhibiting persistent pulmonary sequelae at 12 months of follow-up, and then to investigate which clinical characteristics could predispose to these radiological findings.

## Materials and methods

### Study population and design

In this single-center observational cohort study, 421 patients were consecutively evaluated at the post-COVID-19 clinic of our hospital after discharge. Eligible patients were previously admitted to the Division of Infectious and Tropical Diseases of the University Hospital of Padova from the end of February 2020 until the end of April 2021. Inclusion criteria were: (i) age ≥ 18 years at the moment of hospital admission and (ii) diagnosis of SARS-CoV-2 infection by positive real-time polymerase chain reaction (RT-PCR) on the nasal-pharyngeal swab or on bronchoalveolar lavage (BAL). Exclusion criteria were: (i) pregnancy or breastfeeding status, (ii) having only one or more chest-X-ray (CXR) as a unique radiological investigation, (iii) missing on follow-up visit, or (iv) absence of computed tomography (CT) scan imaging at 12 months. For studying purposes, we completed the recruiting process in April 2021 which allowed the collection of all the data until April 2022, for a global period of 1-year follow-up. During hospitalization, positivity to SARS-CoV-2 was confirmed by a nasal or oropharyngeal swab RT-PCR (12). High-resolution CT (HRCT) was used to evaluate the persistence and characteristics of radiological changes during follow-up visits. Based on the CT changes at 12 months, the whole population was then categorized into two groups: the NOT-RECOVERED group (NOT-REC) when CT still showed lung abnormalities and the RECOVERED group (REC) when CT demonstrated normal lung parenchyma along the follow-up. Symptoms, maximal FiO_2_ (FiO_2_ max.), gas exchange values (PaO_2_/FiO_2_), days of hospital stay, and treatment during hospitalization were collected. Comorbidities were categorized as cardiovascular diseases (CVDs), respiratory diseases, metabolic diseases (including diabetes mellitus, obesity, and dyslipidemia), autoimmune diseases, and oncologic diseases (including lung, prostate, pancreatic, breast, and colon cancer). Based on the level of care, we distinguished those requiring low-intensity medical care (LIMC) and high-IMC (HIMC), as previously described (7). Pulmonary function tests were collected during follow-up visits, indeed the study was planned for two follow-up visits at 6 and 12 months from hospital discharge. Results from 6 months follow-up, as well as inclusion and exclusion criteria and study procedures, are summarized in the manuscript by Cocconcelli et al. (13). The study protocol acts by the ethical guidelines of the 1975 Declaration of Helsinki and was reviewed and approved by the Ethics Committee of the University Hospital of Padova (nr.: 46430/03.08.2020).

### Radiological evaluation

All the CTs were performed by a 64-slice Siemens Somatom Sensation (Siemens Healthcare, Erlangen, Germany), with a slice ≤0.05. Radiological evaluation (REC vs. NOT-REC) was made by two expert radiologists (CG, GF), who were blinded to clinical data and with experience in the evaluation and quantitation of interstitial lung diseases (ILDs) features. After independent evaluation, disagreement between radiologists was resolved by consensus. All the CT images were scored through a composite semi-quantitative scale, as previously described (13). In particular, the extent of ground-glass opacities (GGO), interstitial thickening (IT), and consolidations (CO) was assessed for each lobe using a scale from 0 to 100 and the result was expressed as the mean value of the five lobes for each radiologic feature. The presence or absence of bronchiectasis and curvilinear or linear band opacities for each of the five lung lobes were also evaluated. The level of interobserver agreement was obtained for each patient and expressed as Cohen’s *k* value. For dichotomic parameters (bronchiectasis and band opacities), the patient was considered affected by these abnormalities whenever at least one single lobe was involved.

### Statistical analysis

Continuous variables were described as median and interquartile range (IQR; 25–75), while categorical variables were shown as absolute (*n*) and relative values (%). We used the chi-square test and Fisher’s exact test for categorical variables, while the Mann–Whitney *U* tests were used for continuous variables. Univariable and multivariable logistic regression analyses were performed to detect the factors associated with radiological consequences (NOT-REC) at 12 months. SPSS Software version 25.0 (IBM Corp., New York, NY, USA) was used for all data analysis. We considered a statistically significant *p*-value < 0.05.

## Results

### Baseline characteristics of the entire study population

**FIGURE 1:**
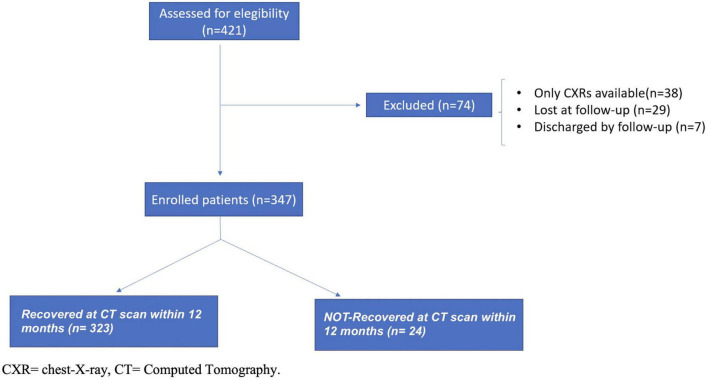
Enrollment flow-chart of patients discharged from hospital and included in the study cohort.

A total of 421 patients started the follow-up evaluation at our post-COVID-19 clinic and were initially considered the study population. At the end of the study at 12 months, 347 patients met inclusion and exclusion criteria and were enrolled in the study (Figure 1). Analysis of the cohort that completed the 12-month period showed that patients were predominantly men (62%) with a median age of 63 years old (53–72 years) and a body mass index (BMI) of 27 (24–30 kg/m^2^), as reported in Table 1. Current smokers were 5%, while non-smokers and former smokers were 63 and 33%, respectively. Patients were predominantly affected by CVDs (50%) and by metabolic diseases (45%). Almost the entire population manifested fever during hospitalization (*n* = 331; 99%), while cough and dyspnea were present in almost half of the patients (54 and 47%; respectively). Regarding treatment during hospitalization, the most administered therapies were heparins (81%) and corticosteroids (69%). After discharge, corticosteroids were prescribed in 56% of patients, as reported in the Supplementary Table 1.

**TABLE 1:** Baseline demographics and clinical characteristics of the overall population evaluated at post-COVID clinic, and of the two subgroups categorized according to the presence or absence of radiological recovery at 12 months.

	Available data	Overall population (*n* = 347)	REC (*n* = 323)	NOT-REC (*n* = 24)	*p*
**Demographic data**					
Male–*n* (%)	347	217 (62)	200 (62)	17 (71)	0.51
Age at admission–years	347	63 (53–72)	63 (53–71)	67 (62–76)	0.02
BMI–kg/m^2^	247	27 (24–30)	27 (25–30)	26 (24–30)	0.23
**Smoking history**					
Pack years	334	0 (0–5)	0 (0–5)	3.1 (0–21)	0.06
Current–*n* (%)	344	16 (5)	12 (4)	4 (17)	0.02
Non-smoker–*n* (%)	344	216 (63)	205 (64)	11 (46)	0.08
Former–*n* (%)	344	114 (33)	105 (33)	9 (37)	0.66
**Comorbidities**					
Cardiovascular diseases–*n* (%)	347	174 (50)	161 (50)	13 (54)	0.83
Respiratory diseases–*n* (%)	347	50 (14)	44 (14)	6 (25)	0.13
Autoimmune diseases–*n* (%)	347	52 (15)	45 (14)	7 (29)	0.07
Metabolic diseases–*n* (%)	347	158 (45)	147 (45)	11 (46)	0.99
Oncologic diseases–*n* (%)	347	57 (16)	52 (16)	6 (21)	0.57
**Hospitalization characteristics**					
FiO_2_ max during hospitalization	337	36 (27–70)	36 (24–66)	75 (32–100)	0.01
Hospitalization–days	347	10 (6–17)	10 (6–16)	17 (10–41)	0.001
High degree of care–*n* (%)	347	69 (20)	57 (18)	12 (50)	0.0006
PaO_2_/FiO_2_ at admission	205	295 (218–342)	295 (223–343)	201 (101–314)	0.01
**Symptoms during hospitalization**					
Fever–*n* (%)	335	331 (99)	289 (92)	22 (100)	0.39
Asthenia–*n* (%)	335	119 (35)	112 (36)	7 (32)	0.70
Dyspnea–*n* (%)	335	158 (47)	140 (45)	18 (75)	0.0008
Anosmia/Ageusia–*n* (%)	335	104 (31)	96 (31)	8 (33)	0.58
Muscular alterations–*n* (%)	335	61 (18)	55 (18)	6 (25)	0.25
Headache–*n* (%)	335	36 (11)	35 (11)	1 (4.2)	0.33
Gastrointestinal–*n* (%)	335	74 (22)	69 (22)	5 (21)	0.94
Cough–*n* (%)	335	182 (54)	168 (54)	14 (58)	0.36

Values are expressed as numbers and (%) or median and interquartile range (IQR), as appropriate. BMI: body mass index. To compare demographics between recovered (REC) and not-recovered (NOT-REC), the chi-square test and Fisher’s *t*-test (*n* < 5) for categorical variables and Mann–Whitney *t*-test for continuous variables were used.

### Baseline characteristics and lung function according to radiological sequelae CT findings at 12 months

**FIGURE 2:**
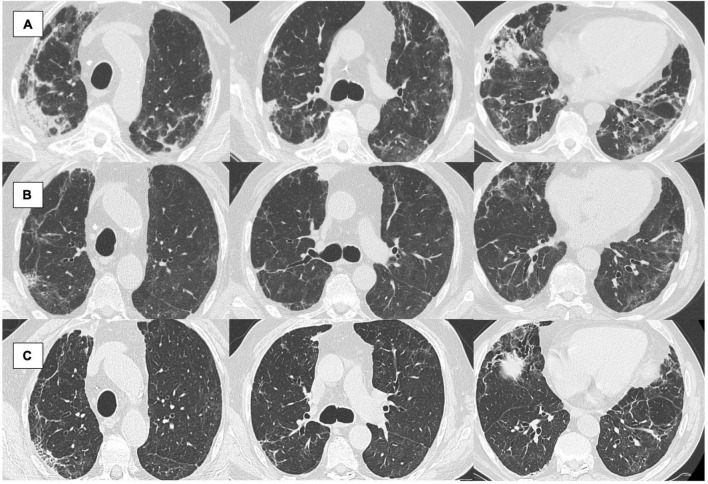
**(A–C)** An 84 years-old male patient followed in our post-COVID-19 clinic. **(A)** Computed tomography (CT) scan at 1 month from hospital admission: diffuse ground-glass opacities (GGO), also with consolidations (CO) in particular in the apical subpleural region and right basal; **(B)** CT at 6 months from hospital admission: ameliorated, GGO persist in the lung bases and the medium lobe; still showing interstitial thickening (IT) with bronchiectasis; **(C)** CT scan at 12 months from hospital admission: sclerosis of the apical subclavian regions, atelectatic thickening in the right upper lobe, diffuse bronchiectasis, and dystelectasis (evaluated through the semi-quantitative grading as follows: 0% of mean GGO extension of the five lobes; 21% of mean IT; 0% CO; 1 bronchiectasis; 1 of linear and curvilinear band opacities).

At the end of the 1-year follow-up, 24 out of 347 patients (6.9%) presented radiological sequelae at 12 months (NOT-REC). Clinical and demographic characteristics of patients divided into REC (*n* = 323) and NOT-REC groups (*n* = 24) are summarized in Table 1. NOT-REC were significantly older [67 (62–76) years vs. 63 (53–71) years; *p* = 0.02] and more frequently current smokers [4 (17%) vs. 12 (4%); *p* = 0.02]. Regarding hospital stay and disease severity, the NOT-REC group displayed significantly worsen parameters compared with the REC group: the median of the maximum FiO_2_ reached during the hospitalization was higher [75% (32–100) vs. 36% (24–66); *p* = 0.01], the median duration of hospitalization was longer [17 (10–41) days vs. 10 (6–16) days; *p* = 0.001], and the median of the PaO_2_/FiO_2_ ratio at admission was lower [201 (101–314) vs. 295 (223–343); *p* = 0.01]. Among all treatments, in the NOT-REC group, other antibiotics (62% vs. 31%; *p* = 0.003) and corticosteroids (87% vs. 67%; *p* = 0.04) were more frequently used during the hospital stay, compared with the REC group (Supplementary Table 1). For all the other drugs, including the administration of corticosteroids after discharge, we did not find any between-group difference (*p* = 0.20). Regarding radiological sequelae, in NOT-REC patients (*n* = 24), the most frequent alteration was IT, which was observed in 21 patients (88%) and with a median extension of 4%. GGO was found in 19 patients (79%) with a median extension of 3.5%, while CO were present in only 2 patients (8%) with an extension of <1% (Figures 2A–C). The linear and curvilinear band opacities were reported in 16 patients (66%) and bronchiectasis in 7 patients (29%), as reported in Table 2. Analyzing pulmonary function tests in the whole population, we observed normal lung volume at first follow-up [Forced Vital Capacity (FVC% pred.): 92% (81–104) and Forced Expiratory Volume in the first second (FEV1% pred.): 95% (84–137)]. Moreover, patients from the NOT-REC group showed similar parameters to patients from the REC group, as reported in Table 3.

**TABLE 2:** Chest high resolution computed tomography (HRCT) scan characteristics of the not-recovered (NOT-REC) group (*n* = 24) at 12-month.

Ground-glass opacities (GGO)	
GGO [*n* (%)]	19 (79%)
Extent of GGO	3.5%
**Interstitial thickening (IT)**	
IT [*n* (%)]	21 (88%)
Extent of IT	4%
**Consolidations (CO)**	
CO [*n* (%)]	2 (8%)
Extent of CO	<1%
**Bronchiectasis**	
Bronchiectasis [*n* (%)]	7 (29%)
**Curvilinear and liner band opacities**	
Curvilinear and liner band opacities [*n* (%)]	16 (66%)

Values are expressed as numbers and (%).

**TABLE 3:** Pulmonary function tests of patients at first follow-up visit according to presence or absence of radiological recovery at 12 months (*n* = 347).

	Overall population (*n* = 347)	REC (*n* = 323)	NOT-REC (*n* = 24)	*p*
FVCabs–liters	3.3 (2.8–4.0)	3.3 (2.8–4.0)	3.2 (2.4–4.6)	0.38
FVC pred–%	92 (81–104)	92 (81–104)	97 (70–103)	0.79
FEV1abs–liters	2.8 (2.3–3.3)	2.8 (2.3–3.3)	2.6 (2.0–3.5)	0.37
FEV1 pred–%	95 (84–137)	95 (84–137)	99 (55–121)	0.74

FVC: forced vital capacity, FEV1: flow expiratory volume at first second. Values are expressed as median and interquartile range (IQR). To compare the pulmonary function test between recovered (REC) and NOT-REC, the Mann–Whitney test for continuous variables was used.

### Predictors of post-COVID-19 pulmonary sequelae

At the univariable analysis, age ≥ 63 years [2.8 OR; 95% CI (1.09–7.33); *p* = 0.03] and being a current smoker [5.2 OR; 95% CI (1.53–17.53); *p* = 0.008] were identified as risk factors for having persistent radiological sequelae at 12 months follow-up after COVID-19 pneumonia. Among hospital stay characteristics, an hospitalization time ≥ 10 days [OR 2.9; 95% CI (1.14–7.61), *p* = 0.03], the high degree of care [4.7 OR; 95% CI (1.99–10.92); *p* = 0.0001]; FiO_2_ max. ≥ 36% [2.6 OR; 95% CI (1.01–6.78); *p* = 0.047] and other antibiotics [3.6 OR; 95% CI (1.54–8.61); *p* = 0.003], resulted as dependent risk factors for having post-COVID-19 pulmonary changes (Table 4). On the multivariable analysis, adjusted for the previous risk factor, we found that smoking history, particularly being a current smoker, was an independent predictor for lung sequelae after 12 months from COVID-19 pneumonia and hospitalization [5.6 OR; 95% CI (1.41–22.12), *p* = 0.01].

**TABLE 4:** Risk factors associated with the persistence of pulmonary sequela in the overall population (*n* = 347).

	Univariable analysis	*p*	Multivariable analysis	*p*
	**OR (95% CI)**		**OR (95% CI)**	
**Demographics**				
Age ≥ 63 years	2.8 (1.09–7.33)	0.03	2.6 (0.96–7.18)	0.06
BMI ≥ 27 kg/m^2^	0.4 (0.15–1.07)	0.07		
**Smoking history**				
Current smoker–yes	5.2 (1.53–17.53)	0.008	5.6 (1.41–22.12)	0.01
No-smoker–yes	2.0 (0.86–4.58)	0.11		
**Comorbidities**				
Cardiovascular disease–yes	1.2 (0.52–2.73)	0.68		
Respiratory disease–yes	2.1 (0.79–5.61)	0.13		
Autoimmune disease–yes	2.5 (0.99–6.48)	0.051		
Metabolic disease–yes	1.0 (0.44–2.33)	0.98		
Oncologic disease–yes	1.4 (0.49–3.84)	0.55		
**Hospitalization characteristics**				
Hospitalization time ≥ 10 days	2.9 (1.14–7.61)	0.03	1.0 (0.25–3.29)	0.89
High degree of care–yes	4.7 (1.99–10.92)	0.0001	2.6 (0.83–8.35)	0.10
FiO_2_ max. ≥ 36%	2.6 (1.01–6.78)	0.047	1.1 (0.31–3.86)	0.89
PaO_2_/FiO_2_ ≥ 295	0.4 (0.14–1.25)	0.12		
**Treatment during hospitalization**				
Hydroxychloroquine/chloroquine–yes	1.4 (0.61–3.24)	0.42		
Azithromycin–yes	0.7 (0.33–1.75)	0.52		
Ceftriaxone–yes	1.1 (0.44–2.38)	0.95		
Other antibiotics–yes	3.6 (1.54–8.61)	0.003	2.4 (0.84–3.28)	0.10
Lopinavir/ritonavir–yes	1.9 (0.74–4.73)	0.18		
Remdesivir–yes	1.4 (0.58–3.24)	0.47		
Other antiviral–yes	2.7 (0.31–24.6)	0.36		
Tocilizumab–yes	2.9 (0.78–10.9)	0.11		
Corticosteroids–yes	3.4 (0.98–11.6)	0.052		
Heparins–yes	0.88 (0.32–2.46)	0.81		
Corticosteroids during follow-up–yes	1.8 (0.74–4.59)	0.19		

BMI: body mass index.

## Discussion

To the best of our knowledge, limited studies have explored the predictive factors of pulmonary sequelae in consecutive patients affected by COVID-19 and with such a long follow-up. In this study, 347 patients were evaluated and we found that 24 (6.9%) subjects still presented radiological changes on CT scans after a 1-year follow-up (NOT-REC group). In line with a previous study (8), these patients were older than those who REC (67 years vs. 63 years; *p* = 0.02), moreover adults older than 63 years showed nearly three times the risk of developing abnormalities on CT scan at 12 months, as shown by univariable analysis. Furthermore, NOT-REC patients had a worse clinical course, compared to patients who REC, during the hospital stay. In fact, the median maximum FiO_2_ required was two-fold higher and the PaO_2_/FiO_2_ ratio was lower in NOT-REC patients. Even if a strong correlation resulted between the severity of the acute illness and the persistence of lung changes, none of the indicators received further confirmation as independent predictors in multivariable analysis. This is in line with previous studies, indeed, it has been reported that patients who presented a more severe acute COVID-19 pneumonia, as indicated by ventilatory support, gas exchange index, and duration of hospital stay, are the same who present radiologic involvement during follow-up visits (at 4, 6, or 12 months) (8–11, 13–15). As confirmed by recent evidence, dyspnea was more frequent at hospital admission in the NOT-REC group, this is a further signal of worse clinical presentation during the acute illness (14). Similar results were recently displayed by Faverio et al. (16) who observed a cohort of 287 patients at 12-month follow-up from hospitalization. The authors showed that fibrotic sequelae at HRCT scans were found in a strict minority of patients (3, 1% of the study cohort), while the so-called “mild non-fibrotic radiological abnormalities” were observed in the majority of cases (66% of the entire cohort) with interstitial lung involvement, particularly GGO and reticular abnormalities, as subpleural curvilinear lines, as the main radiologic pattern (16). Besides, as in our cohort, the anatomical extension of these abnormalities was limited, with a mean lobar involvement that ranges between 13 and 17% of each entire single lobe (16). This report, together with our findings, is in contrast with Tarraso et al. (17) who reported in a multicenter prospective study a higher percentage of patients (23%) with fibrotic-like sequelae after 1 year of follow-up. However, it should be mentioned that the authors did not score the extension of lung lesions on CT, thus limiting the comparison with our assessment (17). When analyzing the main radiological characteristics presented in the NOT-REC group, our findings are in line with Huang et al. (18) that found, at 6 months, a more severe involvement in those patients who required a higher degree of care, with the GGO as the most frequent radiological feature, followed by the irregular lines. Overall, in our study, the lung involvement at 12 months was minimal since the median involvement reached 4% for IT and 3.5% for GGO (with the maximum lung involvement of 21 and 22% in one patient, regarding IT and GGO, respectively). Indeed, it remains to be elucidated if the fibrotic lesions are strictly caused by the infection or if the contribution of mechanical ventilation to lung injury should be considered. Interestingly enough, both the univariable and multivariable analyses confirmed that being an active smoker, at the time of infection, represents an independent predictor for long-term pulmonary sequelae with a five times greater risk regardless of the severity of COVID-19 pneumonia. As very recently summarized by Benowitz et al. (19) smokers resulted in a greater risk of developing severe disease following SARS-CoV-2 infection than non-smokers and the main mechanisms underlying this association might include up-regulation of angiotensin-converting enzyme-2 receptors, immune suppression, oxidative stress, inflammation, and vascular injury. As the pandemic has evolved, important research questions have emerged particularly regarding the so-called post-COVID-19 or long COVID and how these long-term sequelae might be affected by tobacco product use. Within this topic, our finding seems to point out, for the first time, the potential association between cigarette exposure and the persistence of fibrotic-like changes in the lung, following SARS-CoV-2 infection. Interestingly, as previously shown in patients with Idiopathic Pulmonary Fibrosis, cigarette smoking exposure has been shown to impair adaptive humoral and cellular responses, and exaggerate proinflammatory and innate immune responses limiting the physiological tissue damage/repair responses after viral infection (20, 21). Moreover, recently, using a murine model in which animals were exposed to cigarette smoking and subsequently infected with H1N1 influenza virus, the authors have found an exaggerated fibroblastic response with the proliferation of lung fibroblasts providing new insights into the role of smoking in the dysregulation of healing and fibroblastic processes after a respiratory viral infection (22). Thus, we can speculate that active smokers, infected with SARS-CoV-2, could have an increased likelihood of developing a lung fibroblastic response and unsuccessful lung repair. When considering hospital treatment strategy we found that the category of antibiotics resulted in a risk factor for the persistence of lung damage long term even though at univariate analysis and we can speculate that the NOT-REC group included severe patients which needed a wider approach to managing acute COVID-19 pneumonia. For the same reason, during hospitalization, the NOT-REC group received, more frequently than the REC-group, corticosteroids which may characterize the management of critically ill patients during the acute phase. On the other hand, the use of corticosteroids after hospitalization, in our cohort, is not a confounder since the percentage of administration in the two groups was similar. Furthermore, lung function tests were normal, in particular lung volume. Our findings seem in contrast with Jutant’s (8) study, where they found significant differences in the group with fibrotic lesions both for lung volume and diffusing capacity of the lungs for carbon monoxide compared to those without fibrotic lesions. However, Steinbeis demonstrated that, at 12 months, the degree of pulmonary function impairment still correlates with severity during the acute phase, but it improves over time (23). We cannot ignore some limitations of our study. First, the total lung capacity (TLC) and diffusion of lung carbon monoxide (DLCO) were not routinely assessed. DLCO permits the early detection of interstitial lung involvement, however, is not considered a reliable parameter for monitoring patients with pulmonary fibrosis. Indeed DLCO scores were not used as the primary endpoint in clinical studies of new medications for idiopathic pulmonary fibrosis (IPF) (24–26). Moreover, many studies have reported a reduction in DLCO at 3 or 6 months after SARS-CoV-2 infection (14, 20). Besides, at 12 months, Wu et al. (27) showed how it reverted to normality, so the long-term trend is not still clear and needs further clarification. Lastly, this is not a multicenter study, and it is based on data collected in a single hospital, however, the study cohort we were able to prospectively follow is a very large population and truly reflects all in-hospital disease severity.

## Conclusion

After 12 months from hospitalization for COVID-19 pneumonia, fibrotic-like changes on CT are observed in a small percentage of the study population. These radiological sequelae are minimal and do not affect lung function. Finally, being a current smoker, at the time of infection, is an independent predictor of persistent lung changes after 1 year. Further studies are needed to validate these findings.

## Data availability statement

The raw data supporting the conclusions of this article will be made available by the authors, without undue reservation.

## Ethics statement

The studies involving human participants were reviewed and approved by the Ethics Committee of the University Hospital of Padova, *via* Niccolò Giustiniani n.2, 35128 Padova (nr.: 46430/03.08.2020). The Ethics Committee waived the requirement of written informed consent for participation. Written informed consent was obtained from the individual(s) for the publication of any potentially identifiable images or data included in this article.

## Author contributions

MB, MD, NB, EC, and EB conceived the study. MB and MD coordinated data collection, curation, and analyses. NB analyzed the data. MB, MD, SP, and GC performed data collection. CG and GF scored radiography. NB, MB, and MD wrote the manuscript with revision and supervision from AC, SC, PS, MS, and EB. All authors contributed to the article and approved the submitted version.
