# Supplementary material for: Predictors of pulmonary sequelae after COVID-19 pneumonia: A 12-month follow-up study
Source: Front Med (Lausanne). 2023 Feb 2;10:1084002. doi: 10.3389/fmed.2023.1084002 (PMC9932038; doi:10.3389/fmed.2023.1084002)
Supplement: Supplementary file 1 [file Data_Sheet_1.pdf]

Supplementary Material

Predictors of pulmonary sequelae after COVID-19 pneumonia: a 12-month follow-up study.

N. Bernardinello[1†], E. Cocconcelli[1†], C. Giraudo[2], M. Daverio[1], G. Castelli[1], S. Petrarulo[1], M. Bovo[3], G. Fichera[4], S. Cavinato[5]; A. M. Cattelan[5], M. Saetta[1], P. Spagnolo[1], E. Balestro[1]

1. Respiratory Disease Unit, Department of Cardiac, Thoracic, Vascular Sciences, and Public Health, University of Padova and Padova City Hospital, Padova, Italy

2. Department of Medicine, Institute of Radiology, University of Padova and Padova City Hospital, Padova, Italy

3. University of Padova, School of Medicine

4. Pediatric Radiology Unit, University Hospital of Padova, Padova, Italy

5. Division of Infectious and Tropical Diseases, University of Padova and Padova City Hospital, Padova, Italy

† These authors share the first authorship

*Corresponding author: Dr. Elisabetta Balestro (MD, Ph.D.)

Tel: +39 049 8213702; fax: +39 049 8213110.

Email: elisabetta.balestro@aopd.veneto.it

Table S1: treatment during hospitalization of patients who assisted at follow-up in the post-COVID clinic according to CT scan recovery at 12 months (n=347)

	Overall population (n=347)	REC (n=323)	NOT-REC (n=24)	*p*
**Hydroxychloroquine/chloroquine – n (%)**	132 (38)	121 (38)	11 (46)	0.51
**Azithromycin – n (%)**	209 (60)	196 (61)	13 (54)	0.52
**Ceftriaxone – n (%)**	142 (41)	132 (41)	10 (42)	0.99
**Other antibiotics – n (%)**	116 (33)	101 (31)	15 (62)	**0.003**
**Lopinavir/ritonavir – n (%)**	65 (19)	58 (18)	7 (29)	0.18
**Remdesivir – n (%)**	107 (31)	98 (30)	9 (37)	0.50
**Other antivirals – n (%)**	6 (2)	5 (2)	1 (4)	0.35
**Tocilizumab – n (%)**	18 (5)	15 (5)	3 (12)	0.12
**Corticosteroids – n (%)**	238 (69)	217 (67)	21 (87)	**0.04**
**Heparins – n (%)**	281 (81)	262 (81)	19 (79)	0.79
**IgG plasma – n (%)**	67 (19)	62 (19)	5 (21)	0.79
**Corticosteroids at discharge– n (%)**	195 (56)	179 (55)	16 (70)	0.20

Values are expressed as numbers and (%). To compare treatment during hospitalization between recovered (REC) and not recovered (NOT-REC), a chi-square test or a Fisher's t-test (n < 5) for categorical variables was used.
